# A Metabolomics Approach to Unravel *Cricket Paralysis Virus* Infection in Silkworm Bm5 Cells

**DOI:** 10.3390/v11090861

**Published:** 2019-09-16

**Authors:** Luo-Luo Wang, Luc Swevers, Caroline Rombouts, Ivan Meeus, Lieven Van Meulebroek, Lynn Vanhaecke, Guy Smagghe

**Affiliations:** 1 Department of Plants and Crops, Faculty of Bioscience Engineering, Ghent University, 9000 Ghent, Belgium; Luoluo.wang@ugent.be (L.-L.W.); Ivan.Meeus@UGent.be (I.M.); 2 Insect Molecular Genetics and Biotechnology, Institute of Biosciences and Applications, National Centre for Scientific Research “Demokritos”, 15341 Athens, Greece; swevers@bio.demokritos.gr; 3 Laboratory of Chemical Analysis, Department of Veterinary Public Health and Food Safety, Faculty of Veterinary Medicine, Ghent University, 9820 Merelbeke, Belgium; Caroline.Rombouts@UGent.be (C.R.); Lieven.Vanmeulenbroek@UGent.be (L.V.M.); Lynn.Vanhaecke@UGent.be (L.V.); 4 Laboratory of Cell Biology & Histology, Department of Veterinary Sciences, Antwerp University, Faculty of Pharmaceutical, Biomedical and Veterinary Sciences, 2610 Wilrijk, Belgium

**Keywords:** metabolomics, persistent infection, host metabolism, *Cricket paralysis virus*, silkworm

## Abstract

How a host metabolism responds to infection with insect viruses and how it relates to pathogenesis, is little investigated. Our previous study observed that *Cricket paralysis virus* (CrPV, *Dicistroviridae*) causes short term persistence in silkworm Bm5 cells before proceeding to acute infection. In this study, a metabolomics approach based on high resolution mass spectrometry was applied to investigate how a host metabolism is altered during the course of CrPV infection in Bm5 cells and which changes are characteristic for the transition from persistence to pathogenicity. We observed that CrPV infection led to significant and stage-specific metabolic changes in Bm5 cells. Differential metabolites abundance and pathway analysis further identified specific metabolic features at different stages in the viral life cycle. Notably, both glucose and glutamine levels significantly increased during CrPV persistent infection followed by a steep decrease during the pathogenic stages, suggesting that the central carbon metabolism was significantly modified during CrPV infection in Bm5 cells. In addition, dynamic changes in levels of polyamines were detected. Taken together, this study characterized for the first time the metabolic dynamics of CrPV infection in insect cells, proposing a central role for the regulation of both amino acid and carbohydrate metabolism during the period of persistent infection of CrPV in Bm5 cells.

## 1. Introduction

Viruses can have widespread impact on their hosts, yet viral infections are often persistent and nonlethal, making them difficult to understand and quantify [1,2,3]. In fact, regardless of any organism under investigation, there are viruses able to infect that organism, particularly in a persistent way [2,4,5], which raises questions regarding their significance for the host physiology and the possible interactions with infections of other pathogens [6]. Many studies have demonstrated that multiple abiotic and biotic factors can influence the outcome of virus–host interactions and viral pathogenesis [7,8,9], whilst a poorly studied variable is host metabolism [10,11]. As non-living entities, viruses are exclusively dependent on the materials and energy provided by the host metabolism for their replication [10,12]. Viruses have likely evolved to modify the host metabolism for multiple purposes: an increase in free nucleotide pools necessary for rapid viral genome replication, an increase in amino acid production for rapid virion assembly, and an increase in ATP availability for the high energy costs of genome replication and packaging [12]. However, besides facilitating viral replication, alterations in metabolism could potentially also reflect antiviral defense mechanisms [13,14,15]. Hence, the study of metabolism should not be restricted to the simply supply of energy and biosynthetic materials, but should also extend to a better understanding of its role during viral infection and pathogenesis [8,16,17].

Metabolomics, as well as other “omics” technologies, are rapidly advancing research of physiological and pathological processes because of high computational and analytical power [11,18]. In 2006, Munger and co-workers reported the first eukaryotic virus-infected cell metabolomics study of human cytomegalovirus (HCMV) infection [19], in which many metabolic pathways including glycolysis and fatty acid synthesis were specifically modified. Since then, metabolomics has been successfully applied to a variety of virus infections in mammals, plants and insects, and samples involved body fluids or tissues, as well as in vitro persistently and acutely infected cell cultures [19,20,21,22,23]. In all cases, dramatic changes in host cellular metabolism were observed. To date, applications of metabolomics in the field of virology have successfully enabled researchers to measure the highly dynamic host metabolism as induced by virus infection [11] and to identify specific metabolites and pathways involved in viral infection, pathogenesis and the crosstalk with the immune system [8]. 

Previous work from our laboratory described an in vitro system of silkworm (*Bombyx mori*)-derived Bm5 cells that were persistently infected by Cricket Paralysis virus (CrPV; *Dicistroviridae*) for a short term period (2–3 weeks) before transition to pathogenicity, whereas CrPV infection of the fruit fly (*Drosophila melanogaster*)-derived S2 cells evolved much more rapidly and resulted in widespread mortality within 48 h post-infection [24]. This short-term persistence period in Bm5 cells is characterized by low levels of viral replication and virion production along with the production of viral siRNAs (small interfering RNAs), whilst the pathogenic period is preceded by significantly increased virion production and the transcriptional activation of immune-related genes (e.g., genes encoding RNAi factors and transcription factors in the Toll, Imd, and Jak-STAT pathways). The CrPV-Bm5 infection system therefore represents a suitable and accessible model to study the nature of persistent infections and their gradual evolution towards pathogenicity.

In the current study, we applied both a targeted and untargeted metabolomics approach to our in vitro CrPV-Bm5 infection model. We found that CrPV can trigger significant metabolic changes in Bm5 cells which are nevertheless significantly divergent among different infection stages, particularly with respect to central carbon metabolism. Changes in levels of amino acids and carbohydrates are proposed to represent important intrinsic host mechanisms that affect the outcome of CrPV infection and are possibly manipulated by CrPV for optimal replication. Those findings may have implications for the regulation of viral pathogenesis and the development of persistently infected viral culture systems.

## 2. Materials and Methods

### 2.1. Experimental Design

Bm5 cells were maintained at 27 °C in IPL-41 Insect Medium (Sigma Aldrich, Bornem, Belgium) supplemented with 10% fetal bovine serum (FBS), and the CrPV suspension was prepared as previously described [25]. Bm5 cells were infected with CrPV and cellular extracts were prepared at different times after infection. Cellular extracts were processed by UHPLC-Orbitrap-MS for both untargeted and targeted metabolomics. In untargeted metabolomics (“fingerprinting”), all possible metabolites that were differentially present in experimental samples were defined by PCA-X and OPLS-DA modelling. In targeted metabolomics (“profiling”), the differential presence of a collection of approximately 300 known metabolites among experimental samples was determined directly. Because their identity is known, metabolites pathway analysis can be performed in a straightforward manner.

**Figure 1:**
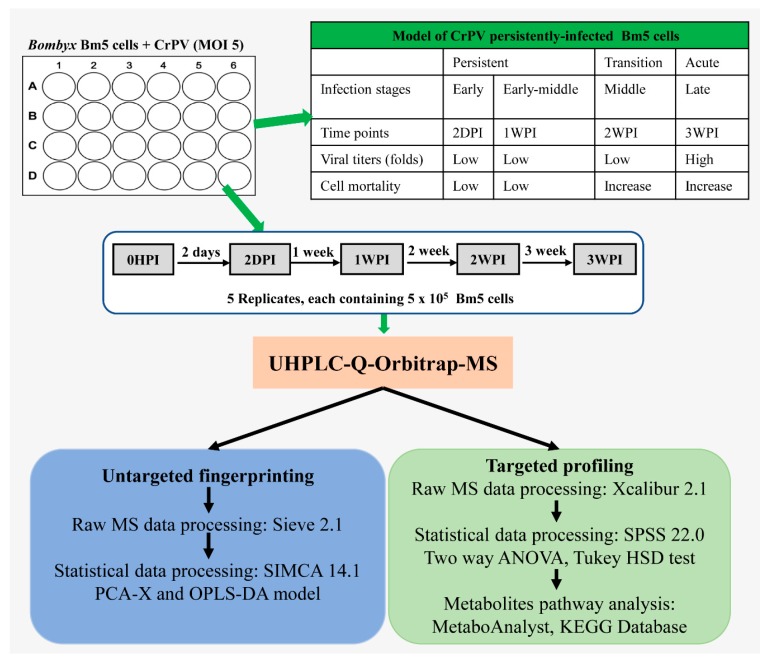
Experimental design and work flow of the metabolomics study. Bm5 cells were infected with CrPV (MOI 5) and cellular extracts (5 replicates) were prepared at different times after infection. Cellular extracts were processed by UHPLC-Orbitrap-MS for both untargeted and targeted metabolomics. In untargeted metabolomics (“fingerprinting”), all possible metabolites that were differentially present in experimental samples were defined by PCA-X and OPLS-DA modelling. In targeted metabolomics (“profiling”), the differential presence of a collection of approximately 300 known metabolites among experimental samples was determined directly. Because their identity is known, metabolites pathway analysis can be performed in a straightforward manner.

As illustrated in Figure 1, Bm5 cells were seeded at 4 × 10^5^/mL in a 12-well plate followed by inoculation with CrPV at multiplicity of infection (MOI) of 5. At the end of every week, Bm5 cells were sub-cultured at 1:1 (*v:v*) with fresh medium. Samples were withdrawn for metabolomics analysis at four time points that correlate with different steps of the viral infection process: Early (2DPI, 2 days post-infection), early–middle (1WPI, 1 week post-infection), middle (2WPI, 2 weeks post-infection) and late (3WPI, 3 weeks post-infection). The sample collection therefore covered the period of persistent infection by CrPV (2DPI, 1WPI), the transition to pathogenic infection with increased mortality (2WPI) and acute infection (3WPI) [24], whereas cellular extracts before infection by CrPV (0HPI) were prepared as control group. At each sampling time point, one well of the 12-well plate was used for quantification of cell numbers. Consequently, the same amount of Bm5 cells (5 × 10^5^ cells) was used for metabolite extraction at each time point. At approximately four hours before extraction, an identical amount of cells was seeded in new wells, making sure that cells at the same confluency were extracted in each sample.

### 2.2. Extraction of Intracellular Metabolites

The medium was gently aspirated from the wells followed by washing of cells twice with 1 mL of ice-cold PBS. After adding 27 μL of internal standard valine-d_8_ (ISTD, 25 ng/μL) (Sigma-Aldrich), 1 mL of 50:50 (*v:v*) methanol:water mixture on ice was immediately added to quench metabolic activity and to extract polar metabolites. Treated cells were collected into a 1.5-mL microfuge tube and briefly sonicated twice for 45 s on ice. The resulting cell suspension was vortexed and centrifuged at 15,000× *g* for 5 min at 4 °C to remove debris. Ultimately, 100 μL supernatant was transferred to a new 1.5-mL microfuge tube, and consecutively dried using a SpeedVac Plus (Thermo Fisher Scientific, San José, CA, USA). All dried samples were re-suspended in 100 μL ultrapure water and transferred to an LC–MS vial with glass insert. All solvents used were of LC-MS grade and obtained from Fisher Scientific or VWR International. Ultrapure water was obtained from a purified-water system (VWR International, Merck, Darmstadt, Germany). 

### 2.3. Ultrahigh Performance Liquid Chromatography Hyphenated to Quadrupole-Orbitrap High-Resolution Mass Spectrometry (UHPLC-Q-Orbitrap-HRMS) Analysis

The UHPLC-Q-Orbitrap-MS method as used in this study was adopted from Vanden Bussche, et al. [26], as optimized by De Paepe et al. [27]. The chromatographic separation was performed with an Ultimate 3000 XRS UHPLC system (Thermo Fisher Scientific). Analysis was performed on a Q-Exactive^TM^ Orbitrap mass analyzer (Thermo Fisher Scientific) that was equipped with a heated electrospray ionization (HESI II) source operating in polarity switching mode. An Acquity UPLC HSS T3 column (1.8 μm, 150 × 2.1 mm) (Waters), kept at 45 °C, was used, to which a binary solvent system consisting of ultrapure water (A) and acetonitrile (B), both acidified with 0.1% formic acid, was applied at a constant flow rate of 0.4 mL/min. A gradient profile with following proportions (*v/v*) of solvent A was applied: 0–1.5 min at 98%, 1.5–7.0 min from 98% to 75%, 7.0–8.0 min from 75% to 40%, 8.0–12.0 min from 40% to 5%, 12.0–4.0 min at 5%, 14.0–14.1 min from 5% to 98%, followed by 4.0 min of re-equilibration. A pool of all extracts (*n* = 25) was used as quality control (QC) samples for instrument conditioning and data normalization. All solvents used were of LC-MS grade. Experimental samples were run in a randomized order (except for QC samples, which were analyzed in duplicate after every ten experimental samples).

### 2.4. Data Analysis

#### 2.4.1. Untargeted Data Analysis

LC-MS raw data were imported into a Sieve 2.1 software package (Thermo Fisher Scientific) for peak extraction and alignment, deconvolution and noise removal. The data for each ionization mode (+ or −) were processed separately during peak list generation to achieve better model characteristics in subsequent multivariate analysis. Parameter settings for identification of chromatographic peaks included frame time width of 0.5 min, *m*/*z* range of 53.4–800 dalton, *m*/*z* width of 6 ppm, retention time of 0.5–16 min and peak intensity threshold of 1,000,000 arbitrary units. Normalization of the amount of analyzed cell material between samples was performed by dividing the abundance of each component by the total ion count (TIC) of the respective sample [28]. The relative abundance of each component was then calculated by dividing the corresponding mean abundance of the two internal QC samples to correct for potential machine drift [26]. To assess the metabolic differences between the samples sets, principal component analysis (PCA) and orthogonal partial least square-discriminant analysis (OPLS-DA) were performed using Simca^TM^ 14.1 (Umetrics, Malmo, Sweden) multivariate statistics software. Model validation was performed after evaluation of quality parameters such as CV-ANOVA (*p*-value < 0.05), permutation testing (*n* = 100), and Q^2^ (>0.5), R^2^Y (>0.5) [27].

#### 2.4.2. Targeted Data Analysis

Xcalibur^TM^ 2.1 (Thermo Fisher Scientific) software was used for the relative quantification of the collection of approximately 300 metabolites (in-house database) that were detected in the samples and identified based on their *m*/*z*-value and retention time. Statistical analysis was performed using SPSS 22.0 (IBM, Chicago, IL, USA). Graphs were plotted using GraphPad Prism v.4.00 (GraphPad, San Diego, CA, USA). To define the biological relevance of significant alterations in metabolites abundance, metabolic pathway construction was pursued using the web-based platform MetaboAnalyst 4.0 (http://www.metaboanalyst.ca/).

## 3. Results 

### 3.1. Global Metabolic Changes in Bm5 Cells Following CrPV Infection 

**Figure 2:**
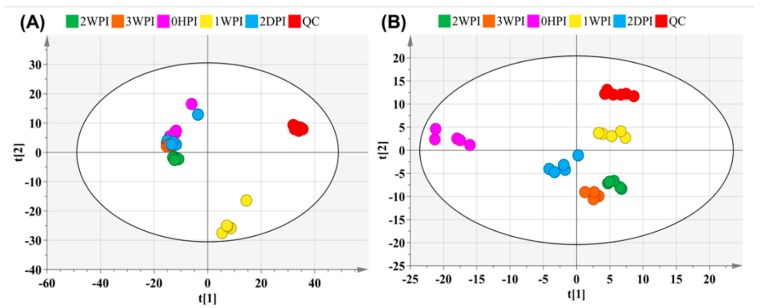
Principal component analysis (PCA)-X score plot of all analyzed samples of Bm5 cells after CrPV infection. The PCA score plot is presented separately for the positive (**A**) and negative ion mode (**B**). The red and other colors represent the internal quality control (QC) and different time points samples, respectively.

An untargeted metabolomics approach was first performed to determine global changes in host cellular metabolism during CrPV infection. Sieve^TM^ 2.1 data preprocessing resulted in 1193 (+ ionization mode) and 560 (– ionization mode) ions in Bm5 cells. The PCA-X score plots displayed tightly clustered QC groups (both positive and negative ionization modes) in Bm5 samples (Figure 2), indicating good analytical reproducibility and reliability of the UHPLC-Q-Orbitrap-HRMS analysis. Furthermore, as summarized in Table 1, a general overview of the metabolic effects of CrPV infection at different time points on Bm5 cells was established based on the discriminant analysis (OPLS-DA) with corresponding datasets. Notably, OPLS-DA analysis clearly discriminated different infection stages of CrPV in Bm5 cells, implicating that the metabolome of Bm5 cells was significantly modified during the course of CrPV infection at different stages (Table 1). All datasets were validated by CV-ANOVA (*p* < 0.01) and the permutation test. 

### 3.2. Metabolites in Bm5 Cells Following CrPV Infection

**Figure 3:**
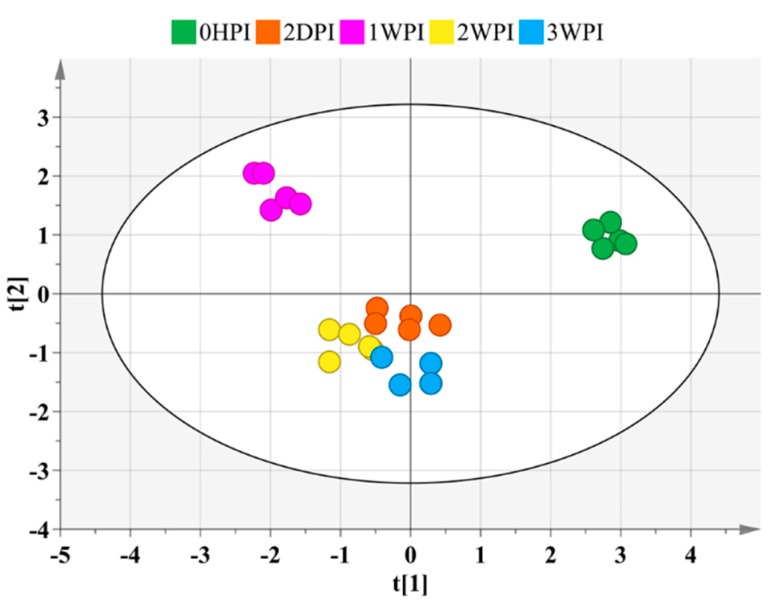
Score plot generated from the PCA-X of targeted metabolites. Mapped are 59 identified targeted metabolites identified at different time points after CrPV infection in Bm5 cells. Samples of different time points are shown in different colors and there is a clear separation among the different experimental conditions.

Among approximately 300 polar metabolites present in our in-house library, 59 metabolites were identified and retained for semi-quantitative metabolic profiling in Bm5 cells (Table S1). These metabolites included 32 amino acids, 14 carbohydrates, 5 carboxylic acids, and 8 compounds from other chemical classes. OPLS-DA analysis with those identified metabolites revealed clear separation between different stages post CrPV infection (R^2^X = 90.4%, R^2^Y = 97.8%, Q^2^ = 93.6%) (Figure 3). 

**Figure 4:**
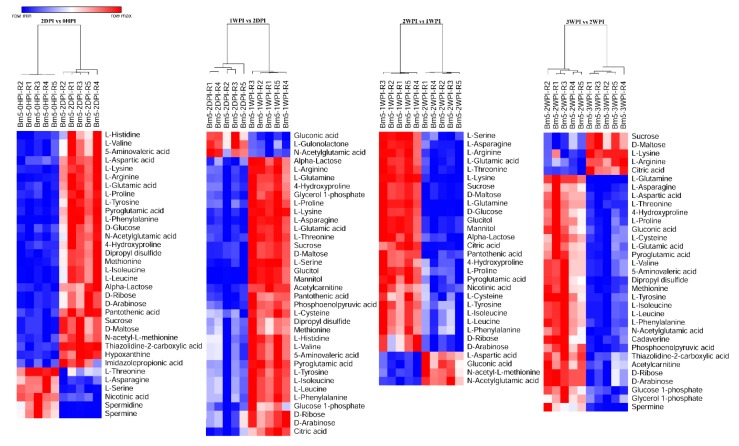
Heat maps of differential levels of metabolites at different time points after CrPV infection: Comparison between consecutive stages of infection. Hierarchical clustering was used to separate individual samples (X-axis). The Y-axis represents individual metabolites that were identified and differentially expressed with respect to preceding and following time points. The profile of targeted metabolites showed a clear separation among consecutive stages at different time points after CrPV infection in Bm5 cells. Normalized signal intensities are visualized as a color spectrum in the heat maps. Red represents high expression, and blue represents low expression of dysregulated metabolites. Different heat maps compare different time points after CrPV infection (5 repeats of each).

As can be seen in Table 2, CrPV infection resulted in significant changes of 34 (58%), 45 (76%), 42 (71%), and 43 (73%) metabolites in the 2DPI, 1WPI, 2WPI, and 3WPI samples, respectively, as compared to the control group 0HPI by multiple T test (*p* < 0.05). To further observe patterns of metabolite abundance overall, we performed heat map analysis which clearly displayed that in CrPV-infected Bm5 cells the majority of the significantly altered metabolites increased at all infection stages as compared to the control (OHPI; Figure S1). The increase, however, occurred mostly during the persistent stages (2DPI and 1WDPI) since it was observed that most metabolites decreased again during the transition from 1WPI to 2WPI and from 2WPI to 3WPI (Figure 4). These results confirmed that the metabolomic profiles of different CrPV infection stages differed significantly in Bm5 cells, a difference that was especially pronounced for the abundance of amino acids and carbohydrates. 

### 3.3. Modified Metabolic Pathways in CrPV-Infected Bm5 Cells

To gain further insight in the nature of persistent infections and the transition to pathogenicity, the pathway analysis was performed with respect to the significantly changed metabolites at each time point. Although a total of 33 pathways was identified in Bm5 cells as being altered during CrPV infection, only 3–4 pathways were identified as having statistical significance (FDR < 0.05) between different time points (Table 3). The three pathways mostly affected by infection over all four time points were: “aminoacyl-tRNA biosynthesis”, “arginine and proline metabolism” and “valine, leucine and isoleucine biosynthesis”. The pathway “alanine, aspartate and glutamate metabolism” was uniquely affected at the 1WPI (Table 3). In addition, the pathway “glutathione metabolism” was significantly altered between the 2WPI and 3WPI samples. With one exception, all pathways in Bm5 cells were related to amino acid metabolism, either for degradation towards feeding into other pathways (e.g., tricarboxylic acid cycle (TCA) cycle), or for biosynthesis to function as building blocks for protein synthesis.

### 3.4. Differential Abundance Analysis of Identified Metabolites

**Figure 5:**
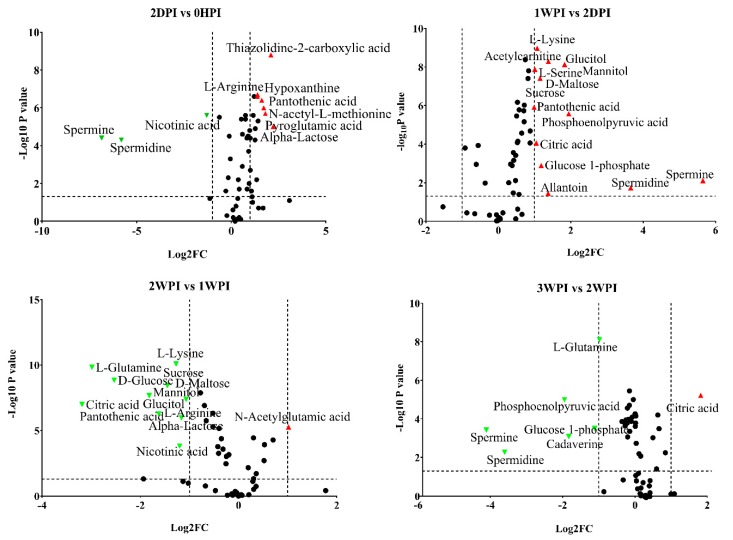
Volcano plots of all identified metabolites in Bm5 cells at different time points of CrPV infection: Comparison between consecutive stages of infection. In the volcano plot, the log scaled value of the fold change (FC, *x*-axis) is plotted against the –log 10 *p*-value from multiple *t*-test analysis (*y*-axis). The dashed lines delineate metabolites with fold change > 2 and *p*-value < 0.05. Compound identities are displayed for both upregulated (right side; red upward triangle) or downregulated (left side; green downward triangle) metabolites. Different volcano plots compare different time points after CrPV infection (5 repeats of each).

**Figure 6:**
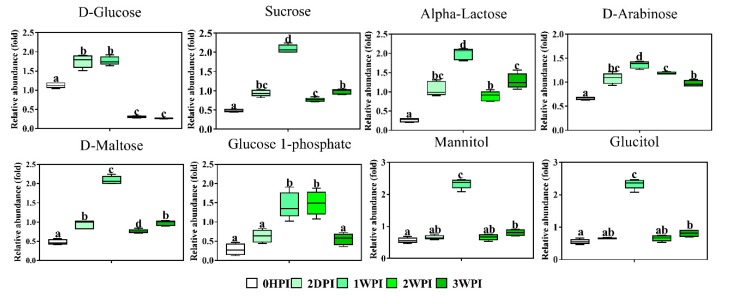
Abundance analysis of the carbohydrates identified in Bm5 cells from different CrPV infection stages. The data shown are mean ± SE (*n* = 5). Different letters indicate significant differences (*p* < 0.05, one-way ANOVA followed by Tukey’s HST test).

**Figure 7:**
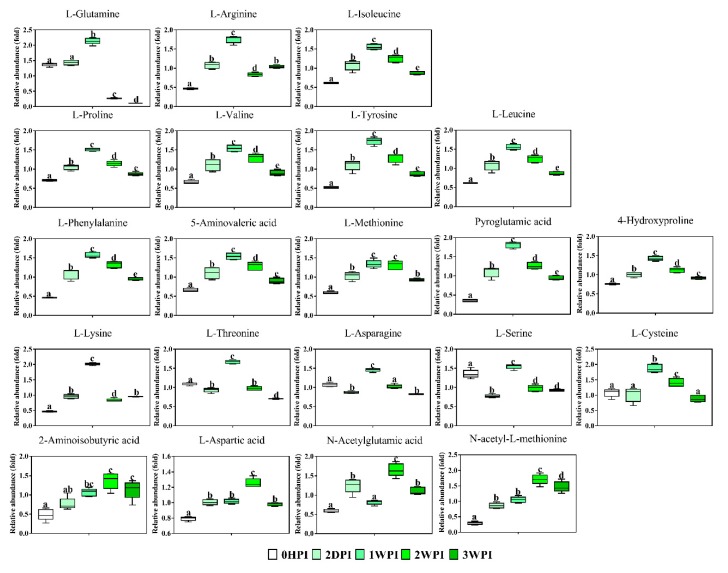
Abundance analysis of the amino acids identified in Bm5 cells from different CrPV infection stages. The data shown are mean ± SE (*n* = 5). Different letters indicate significant differences (*p* < 0.05, one-way ANOVA followed by Tukey’s HST test).

Since 57.6–76.3% of detected metabolites were significantly changed in Bm5 cells during the course of CrPV infection, volcano plots (significance versus fold-change) were generated to identify the most significantly changed metabolites at different time points that contribute to metabolic profile separation. As can be seen in Figure 5 and Figure S2, metabolites that satisfy the condition of *p* < 0.05 and fold change > 2, such as some carbohydrates (e.g., glucose), amino acids (e.g., L-glutamine), spermine, spermidine, citric acid, phosphoenolpyruvic acid, nicotinic acid and pantothenic acid, were further explored. A more detailed analysis of changes in the levels of eight significantly differential expressed carbohydrates is presented in Figure 6, illustrating that all increased in abundance during the persistent infection, but then they decreased during acute infection. The most predominant decrease during the acute infection period was observed for glucose. Analysis was also performed on the abundance of 21 key amino acids in Bm5 cells (Figure 7). Strikingly, except for 2-aminoisobutyric acid, N-acetylglutamic acid, L-asparagine and N-acetyl-L-methionine for which the highest expression level was detected at 2WPI, all other 17 amino acids reached their highest level at 1WPI, after which a decline was observed to levels similar to uninfected cells. Changes in the major amino acids in Bm5 cells were therefore compatible with an increase in protein building blocks (amino acids) during early stages (2DPI and 1WPI) and their incorporation in viral proteins during high rates of virion production at late stages (2WPI and 3WPI). A major divergence in this pattern, however, was observed for glutamine, of which the decline during late stages was much more severe than for the other amino acids (Figure 7).

### 3.5. Key Metabolites during CrPV Infection in Bm5 Cells

Specific assessment of some key metabolites that are required for viral life cycle will provide a deeper understanding of virus replication needs and the transition from persistence to pathogenicity.

#### 3.5.1. Glucose and Glutamine

These metabolites represent two main carbon sources used by eukaryotic cells for the maintenance of energetic and biosynthetic needs. Here, a striking observation relates to the changes in the abundance of glutamine and glucose that become limiting during acute infection (2WPI and 3WPI) in Bm5 cells, when CrPV starts to replicate at high levels and the transition to pathogenicity is reached. Among all metabolites, both glucose (Figure 6) and glutamine (Figure 7) display the most dynamic changes during the course of infection, with significant increases during the persistent infection followed by a sharp decrease at acute infection stages. 

#### 3.5.2. Citric Acid

**Figure 8:**
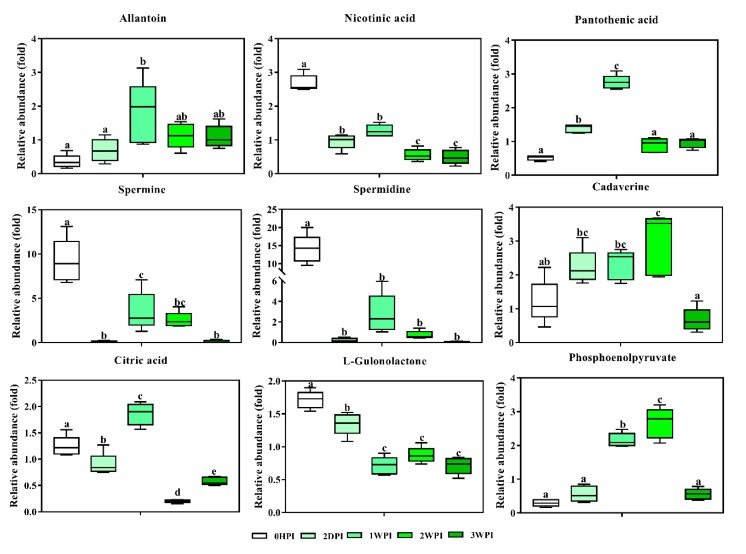
Abundance analysis of key metabolites identified in Bm5 cells from different CrPV infection stages. The data shown are mean ± SE (*n* = 5). Different letters indicate significant differences (*p* < 0.05, One-way ANOVA followed with Tukey’s HST test).

As an important intermediate in the TCA cycle, citric acid is present in the metabolome of all aerobic organisms. Consistent with the existence of different CrPV infection stages in Bm5 cells, citric acid levels showed a dynamic pattern (Figure 8), with significantly lower levels at 2DPI, maximal abundance at 1WPI, a steep decrease at 2WPI, and an increase again at 3WPI. While citric acid is the only detected TCA intermediate here, its importance in the TCA cycle is well-known and involves the coordination between energy production and biosynthesis [29].

#### 3.5.3. Phosphoenolpyruvate

This metabolite has a high-energy phosphate bond and is involved in both glycolysis (production of ATP) and gluconeogenesis. Phosphoenolpyruvate levels increased during the early to middle stages, but became depleted during the late stages (Figure 8).

*Polyamines.* As relatively small molecules, polyamines have been consistently associated with viral infection due to their abundance within the cell and their importance for nucleotide charge neutralization [30]. Here, we observed a steep decline of two key polyamines, spermidine and spermine, during the early stages of CrPV infection in Bm5 cells, followed by partial recovery at the early-middle and middle stages (Figure 8). In contrast, cadaverine, a diamine, was significantly increased during most infection stages (Figure 8).

#### 3.5.4. Vitamins

From our data, three vitamin-related metabolites were present for which significant changes could be observed. Pantothenic acid (vitamin B5) is an essential component of coenzyme-A (CoA), involved in the synthesis of proteins, carbohydrates and fatty acids, and its abundance followed the pattern of most amino acids and carbohydrates. On the other hand, both nicotinic acid (vitamin B3) and L-gulonolactone (metabolite needed for vitamin C synthesis) were significantly decreased during all infection stages in Bm5 cells. 

#### 3.5.5. Purine and Pyrimidine Metabolism

The end-product of pyrimidine metabolism, 2-aminoisobutyric acid (a non-protein amino acid), accumulated gradually as CrPV infection progressed until 2WPI, after which a small decrease was observed (Figure 7). Allantoin, a product of purine metabolism, followed the general pattern of changes in abundance of amino acids and carbohydrates (peak at 1WPI; Figure 8).

## 4. Discussion 

The mechanisms for establishing persistent viral infections and their potential to evolve into pathogenesis are poorly understood. With our previously established model of CrPV-Bm5 infection [24], we have now investigated how a host metabolism is specifically altered during the course of CrPV infection in Bm5 cells and which changes are characteristic for the transition from persistence to pathogenicity. With a dual approach of targeted and untargeted metabolomics, we have shown that CrPV infection leads to significant and stage-specific metabolic changes in Bm5 cells, demonstrating that the UHPLC-Orbitrap-MS system can successfully identify and distinguish the metabolic profiles of Bm5 cell samples at different phases of CrPV infection. Since CrPV first persists at low levels but eventually will replicate to high titers and cause mortality in Bm5 cells, it can be hypothesized that cellular metabolism must be dramatically modified during the course of infection to provide sufficient energy and materials for viral replication and virion production.

Following the observation that CrPV can cause diverse metabolic effects in Bm5 cells, further association of specific cellular metabolic features with different stages of the viral life cycle can provide a deeper understanding of virus replication demands that precede the transition from persistence to pathogenicity. Our results clearly revealed robust metabolic responses in Bm5 cells during the course of CrPV infection, where an average of 70% of detected metabolites (58–76% range) was significantly altered over CrPV infection. Overall, the pattern of accumulation of metabolites, which mostly include amino acids and carbohydrates, can be divided in two periods which correspond broadly to the stage of persistence (2HDPI and 1WPI) and the transition to pathogenicity (2WPI and 3WPI) (Figure 6 and Figure 7).

Given the similar tendency of the increase for nearly all identified amino acids and carbohydrates (with a peak at 1WPI), the data may indicate the establishment of a higher energy status in infected Bm5 cells during the persistence period, that is consistent with an increased maintenance cost for viral replication and/or higher energy needs to maintain normal cell function and survival [16,31]. When the pathway analysis was performed, it was observed that three pathways—including aminoacyl-tRNA (AA-tRNA) biosynthesis, arginine and proline metabolism, and valine, leucine, and isoleucine biosynthesis—were mostly affected by the infection at all time points in Bm5 cells (Table 3). Both the activation of the AA-tRNA pathway and the increase in levels of glutamine, that acts as an important fuel in the cells (Figure 7), indicate the enhancement of protein metabolism in CrPV-infected Bm5 cells during the period of persistence [32]. Furthermore, the other two significantly affected pathways, arginine and proline metabolism and valine, leucine, and isoleucine biosynthesis, are also closely related to protein metabolism. As such, these results provide evidence that in persistently infected Bm5 cells there is an increase in amino acids concentration levels that may be derived from increased biosynthesis or increased host protein catabolism and that can be used for feeding other pathways (e.g., the TCA cycle) or for viral protein synthesis. Such “re-channelization” of the use of amino acids has also been reported during metabolome studies following starvation in insects [32]. 

In our previous study, we found an approximate 1000-fold increase of CrPV titers in the culture medium during the third week of infection, suggesting an efficient CrPV virion production in Bm5 cells. Here it is observed that both amino acids and carbohydrates decrease in cellular abundance during this period (2WPI and 3WPI) (Figure 6 and Figure 7), possibly indicating their consumption during the high energy and biosynthetic demands during the period of acute infection. The most striking changes were observed for both glucose (Figure 6) and glutamine (Figure 7), two major carbon sources for supporting energetic and biosynthetic requirements [31]. Furthermore, it can be proposed that the low levels of glucose and glutamine will contribute to the increased mortality during the later phases of infection.

In contrast to amino acids and carbohydrates, the building blocks for CrPV mRNA or RNA genome synthesis, such as cytosine, thymidine and uracil that are in the list of targeted metabolites (in-house database), were not detected in Bm5 cell samples. Ribose, on the other hand, was detected but it did not show significant changes during CrPV infection. These data may suggest that the building blocks for nucleic acid synthesis are present at low levels in Bm5 cells (in contrast to carbohydrates and amino acids) and do not need further increase to accommodate the needs of CrPV infection. However, a more comprehensive profiling of substances related to nucleic acid metabolism needs to be performed to confirm this. Several other studies have reported that viruses can modify specific metabolic pathways for efficient replication to continue their life cycle [10,29,33,34]. For instance, glycolysis, glutaminolysis, and fatty acid synthesis pathways each show stage-specific requirements for optimal Kaposi’s sarcoma-associated herpesvirus (KSHV) production [33].

It was also noticed that two polyamines, namely spermidine and spermine, were sharply decreased in Bm5 cells at all time points of CrPV infection, particularly at 2DPI (Figure 8). A role for polyamines as important host factors for virus infection is supported by several other studies [30,35,36,37,38]. Dysregulation of cellular polyamines has been associated with various pathological conditions including viral infections [30]. Moreover, several studies indicated a significant role for polyamines in the replication of RNA viruses [36,37,38]. For example, treatment of BHK21 cells with a specific inhibitor of polyamine synthesis resulted in a steep reduction of viral titers, which was efficiently rescued by exogenous polyamines [37]. Increase in polyamines therefore seems to be associated with the stimulation of viral replication (transition to pathogenicity), while depletion occurs during advanced pathogenesis and cell mortality.

With respect to increased energy requirements, increases in citric acid (an essential metabolite in the TCA cycle) [29] and phosphoenolpyruvate (an important energy donor during glycolysis) [39] were also observed during the persistence period (Figure 8). Increases in synthesis were also documented by the change in abundance of pantothenic acid, a component of coenzyme A that is essential for a multitude of enzymatic reactions involving amino acids, carbohydrates, and lipids [40] (Figure 8).

Many studies have demonstrated that multiple abiotic and biotic factors can influence the outcome of virus–host interactions, including the viral life cycle and the physiological status of the infected cell [7,16]. In the context of virus–host cell interactions, as a highly dynamic system, host cells can alter their intracellular metabolism and metabolic activities, with the aim of controlling viral infection; alternatively, viruses can induce changes in cellular metabolism to their advantage [14,15]. For successful replication, each virus species may induce a unique metabolic phenotype in different cell types [12,16]. It is noted that *Bombyx* Bm5 cells that are derived from ovarian tissue, are more resilient to infection than other tissues (e.g., hemocytes, fat body, midgut) [41]. For instance, in *Drosophila*-derived S2 cells, the process of pathogenesis is initiated immediately after CrPV infection and production of large numbers of virions in conjunction with widespread cell mortality occurs within a time frame of 24 to 48 h. Hence, given the different infection kinetics between S2 (derived from hemocytes) and Bm5 cells, it can be assumed, first, that CrPV can alter host-specific metabolic processes in Bm5 cells to create an optimal environment for viral replication, and second, that virus-induced alterations of host metabolism will probably proceed with different dynamics in Bm5 versus S2 cells. Additional future metabolomics analysis of CrPV-infected S2 cells would be an interesting experiment to correlate different infection kinetics in Bm5 and S2 cells with different effects of viral infection on cellular metabolism.

One limitation in this study is that parallel control samples were not included. The differences in cell confluency at each time point, even though strictly controlled, could therefore be a variable that influences the abundance of metabolites. It should also be stressed that functional studies are required to validate the dependence of CrPV infection on host metabolism and to confirm requirements of specific metabolites for optimal CrPV replication. More specifically, additional experiments are required to identify unknown components from untargeted metabolomics that may serve as important host factors during CrPV infection. In this respect, it can be noted that the implemented metabolomics strategy particularly focused on the polar fraction of the metabolome. Thus, application of other “omics” approaches (transcriptomics, proteomics, lipidomics) will be necessary to acquire a more complete picture of the mechanisms by which CrPV can re-program the cellular environment of Bm5 cells (concomitant with resisting the immune response) during the persistence period before robust (acute) replication can be achieved.

In summary, a dual targeted and untargeted metabolomics was employed on CrPV persistently-infected Bm5 cells to investigate how CrPV manipulates Bm5 metabolism during short term persistence and how virus-induced metabolic changes can lead to CrPV pathogenesis. Our data revealed the important changes in host metabolism during CrPV pathogenesis and pointed to several metabolic characteristics during CrPV persistent infection. The general pattern that appears is that Bm5 cells are initially relatively refractory to CrPV infection but that cellular metabolism is gradually altered until favorable conditions for acute (pathogenic) infection are created. Further comparison with metabolome kinetics in the acute CrPV-S2 infection model should be able to clarify this point. Our study also demonstrates, in addition to the importance of glucose and glutamine, that polyamines may play a critical role in CrPV persistence and replication.

## Figures and Tables

**Table 1:** Classification dataset composition and specification of constructed orthogonal partial least square-discriminant analysis (OPLS-DA) models with output of model validation.

Model	Numbers of Instances	Numbers of Model Components (t_o_ + t_p_) ^a^	Model Characteristics ^b^	Cross-Validated ANOVA ^c^	Permutation ^d^
0HPI vs. 2DPI	10	1 + 1 + 0	R^2^Y = 0.993; Q^2^ = 0.973	3.96 × 10^−4^	Good
0HPI vs. 1WPI	10	1 + 0 + 0	R^2^Y = 1.000; Q^2^ = 0.995	6.16 × 10^−9^	Good
0HPI vs. 2WPI	10	1 + 0 + 0	R^2^Y = 0.99; Q^2^ = 0.984	5.52 × 10^−7^	Good
0HPI vs. 3WPI	10	1 + 0 + 0	R^2^Y = 0.996; Q^2^ = 0.987	2.58 × 10^−7^	Good
2DPI vs. 1WPI	10	1 + 0 + 0	R^2^Y = 0.996; Q^2^ = 0.988	2.15 × 10^−4^	Good
1WPI vs. 2WPI	10	1 + 0 + 0	R^2^Y = 0.998; Q^2^ = 0.995	1.03 × 10^−8^	Good
2WPI vs. 3WPI	10	1 + 1 + 0	R^2^Y = 0.998; Q^2^ = 0.958	1.26 × 10^−3^	Good
2DPI vs. 2WPI	10	1 + 2 + 0	R^2^Y = 0.999; Q^2^ = 0.970	3.79 × 10^−3^	Good
2DPI vs. 3WPI	10	1 + 2 + 0	R^2^Y = 1.000; Q^2^ = 0.923	2.03 × 10^−2^	Good
1WPI vs. 3WPI	10	1 + 0 + 0	R^2^Y = 0.999; Q^2^ = 0.994	1.54 × 10^−8^	Good

**^a^** with t_o_ the orthogonal and t_p_ the predictive component; **^b^** with R^2^Y the variation in Y that is explained by the model, and Q^2^ the predictive ability of the model with Q^2^ > 0.5 indicates good model quality; **^c^** a cross-validated ANOVA with *p* value < 0.05 indicates good model quality; **^d^** good permutation testing is achieved if R^2^Y and Q^2^ values of the models based on the permutated data are significantly lower than those based on the real data set.

**Table 2:** Peak abundance ratio of identified metabolites in CrPV-infected Bm5 cells.

	2DPI	1WPI	2WPI	3WPI
***Amino Acids***				
L-Arginine	1.08	1.75	0.84	1.05
L-Asparagine	0.87	1.46	1.02	0.82
L-Aspartic acid	1.00	1.01	1.26	0.98
L-Cysteine	1.02	1.87	1.41	0.88
L-Glutamic acid	0.96	1.70	1.05	0.85
L-Glutamine	1.43	2.14	0.27	0.11
L-Histidine	0.94	1.25	1.24	1.11
L-Isoleucine	1.08	1.55	1.25	0.87
L-Leucine	1.08	1.55	1.25	0.87
L-Lysine	0.96	2.02	0.84	0.95
L-Methionine	1.03	1.34	1.32	0.93
L-Phenylalanine	1.08	1.58	1.33	0.96
L-Proline	1.05	1.51	1.15	0.87
L-Serine	0.77	1.55	0.98	0.93
L-Threonine	0.93	1.67	0.98	0.70
L-Tyrosine	1.11	1.73	1.29	0.87
L-Valine	1.11	1.54	1.29	0.90
N-acetyl-Methionine	0.86	1.05	1.71	1.47
N-acetyl-Glutamic acid	1.25	0.82	1.66	1.11
Pyro-Glutamic acid	1.11	1.81	1.26	0.95
4-Hydroxyproline	1.00	1.42	1.12	0.91
5-Aminovaleric acid	1.11	1.54	1.29	0.90
2-Amino-isobutyric acid	0.77	1.07	1.37	1.14
***Carbohydrates***				
D-Arabinose	1.08	1.37	1.19	0.98
D-Glucose	1.75	1.77	0.31	0.27
D-Maltose	0.94	2.09	0.77	0.98
D-Ribose	0.94	2.09	0.77	0.98
Alpha-Lactose	1.08	1.99	0.89	1.28
Glucitol	0.65	2.34	0.66	0.81
Mannitol	0.65	2.34	0.66	0.81
Gluconic acid	1.29	0.87	1.26	0.92
Glucose-1-phosphate	0.63	1.44	1.49	0.56
Sucrose	0.94	2.09	0.77	0.98
***Other metabolites***				
Allantoin	0.69	1.80	1.12	1.09
Citric acid	0.90	1.86	0.20	0.58
Dipropylsulfide	1.03	1.38	1.33	0.93
Gamma-amino-butyric acid	0.86	1.27	1.57	1.01
Glycerol-1-phosphate	0.75	1.24	1.25	1.02
L-Gulunolactone	1.35	0.72	0.87	0.71
Imidazolepropionic acid	1.46	1.60	1.60	1.35
Nicotinic acid	0.96	1.27	0.55	0.49
Pantothenic acid	1.39	2.75	0.89	0.96
Phosphoenolpyruvic acid	0.56	2.16	2.67	0.55
Thiazolidine-2-carboxylic acid	1.37	1.07	1.53	1.02
Hypoxanthine	1.16	1.12	1.28	1.26
Pipecolic acid	0.61	0.96	0.89	0.80
Spermidine	0.22	2.77	0.73	0.05
Spermine	0.07	3.52	2.54	0.12
Acetylcarnitine	0.54	1.42	1.38	0.97

Red and blue colors represent a significant (*p* < 0.05) increase and decrease in abundance, respectively (compared to uninfected cells). Values represent normalized abundance levels for each metabolite.

**Table 3:** Major metabolic pathways modulated during CrPV infection of Bm5 cells.

Comparison	Pathway	hits	Holm Adjusted P	FDR	Impact
2DPI vs. 0HPI	Aminoacyl-tRNA biosynthesis	15	4.38 × 10^−9^	4.38 × 10^−9^	0.14
	Arginine and proline metabolism	7	5.25 × 10^−3^	2.66 × 10^−3^	0.42
	Valine, leucine and isoleucine biosynthesis	4	3.27 × 10^−2^	1.12 × 10^−2^	1.00
1WPI vs. 0HPI	Aminoacyl-tRNA biosynthesis	16	7.76 × 10^−8^	7.76 × 10^−8^	0.00
	Arginine and proline metabolism	8	5.45 × 10^−3^	2.76 × 10^−3^	0.42
	Valine, leucine and isoleucine biosynthesis	4	1.07 × 10^−1^	3.67 × 10^−2^	1.00
	Alanine, aspartate and glutamate metabolism	5	1.41 × 10^−1^	3.67 × 10^−2^	0.73
2WPI vs. 0HPI	Aminoacyl-tRNA biosynthesis	15	2.44 × 10^−7^	2.44 × 10^−7^	0.14
	Arginine and proline metabolism	9	3.19 × 10^−4^	1.61 × 10^−4^	0.42
	Valine, leucine and isoleucine biosynthesis	4	8.06 × 10^−2^	2.76 × 10^−2^	1.00
3WPI vs. 0HPI	Aminoacyl-tRNA biosynthesis	16	5.05 × 10^−8^	5.05 × 10^−8^	0.14
	Arginine and proline metabolism	8	4.53 × 10^−3^	2.29 × 10^−3^	0.42
	Valine, leucine and isoleucine biosynthesis	4	9.78 × 10^−2^	3.35 × 10^−2^	1.00
1WPI vs. 2DPI	Aminoacyl-tRNA biosynthesis	16	2.69 × 10^−9^	2.69 × 10^−9^	0.14
	Valine, leucine and isoleucine biosynthesis	4	5.35 × 10^−2^	2.71 × 10^−2^	1.00
2WPI vs. 1WPI	Aminoacyl-tRNA biosynthesis	14	2.22 × 10^−8^	2.22 × 10^−8^	0.14
	Arginine and proline metabolism	6	3.14 × 10^−2^	1.59 × 10^−2^	0.37
3WPI vs. 2WPI	Aminoacyl-tRNA biosynthesis	15	4.38 × 10^−9^	4.38 × 10^−9^	0.0
	Arginine and proline metabolism	7	5.25 × 10^−3^	2.66 × 10^−3^	0.37
	Valine, leucine and isoleucine biosynthesis	4	3.27 × 10^−2^	1.12 × 10^−2^	1.00
	Glutathione metabolism	5	6.10 × 10^−2^	1.50 × 10^−2^	0.12

Pairwise metabolite pathway analysis was conducted on the identified metabolite contents between different time points after CrPV infection. Pathways with the most hits, highest Holm adjusted *p*-value (FDR < 0.05), and highest pathway impact values are considered the most significantly affected pathways.

## Supplementary Materials

The following are available online at https://www.mdpi.com/1999-4915/11/9/861/s1, Figure S1: Heat maps of differential levels of metabolites at different time points after CrPV infection: Comparison with uninfected cells, Figure S2: Volcano plots of all identified metabolites in Bm5 cells at different time points of CrPV infection: Comparison with uninfected cells, Table S1: List of identified metabolites in silkworm-derived Bm5 cells upon CrPV infection.
