# Supplementary material for: A Metabolomics Approach to Unravel Cricket Paralysis Virus Infection in Silkworm Bm5 Cells
Source: Viruses. 2019 Sep 16;11(9):861. doi: 10.3390/v11090861 (PMC6784103; doi:10.3390/v11090861)
Supplement: Supplementary file 1 [file viruses-11-00861-s001.pdf]

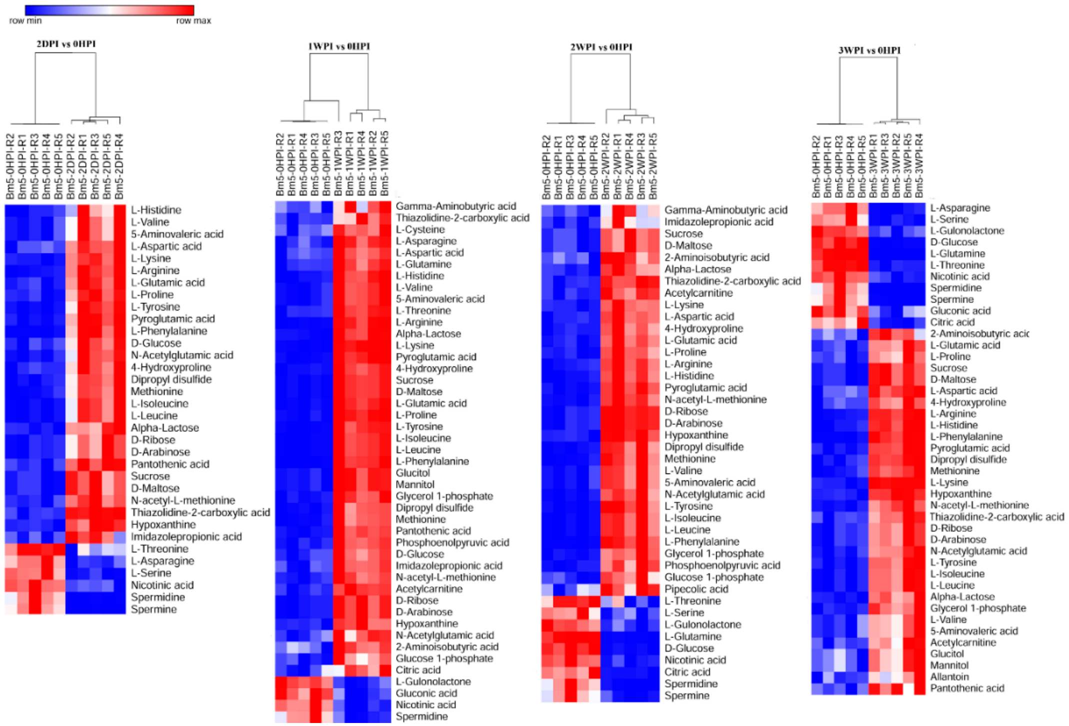

Figure S1. Heat maps of differential levels of metabolites at different time points after CrPV infection: Comparison with uninfected cells. Hierarchical clustering was used to separate individual samples (X-axis). Y-axis represents individual metabolites that were identified and differentially expressed with respect to uninfected cells. The profile of targeted metabolites showed a clear separation from uninfected cells at different time points after CrPV infection in Bm5 cells. Normalized signal intensities are visualized as a color spectrum in the heat maps. Red represents high expression whereas blue represents low expression of dysregulated metabolites. Different heat maps compare different time points after CrPV infection (5 repeats of each).

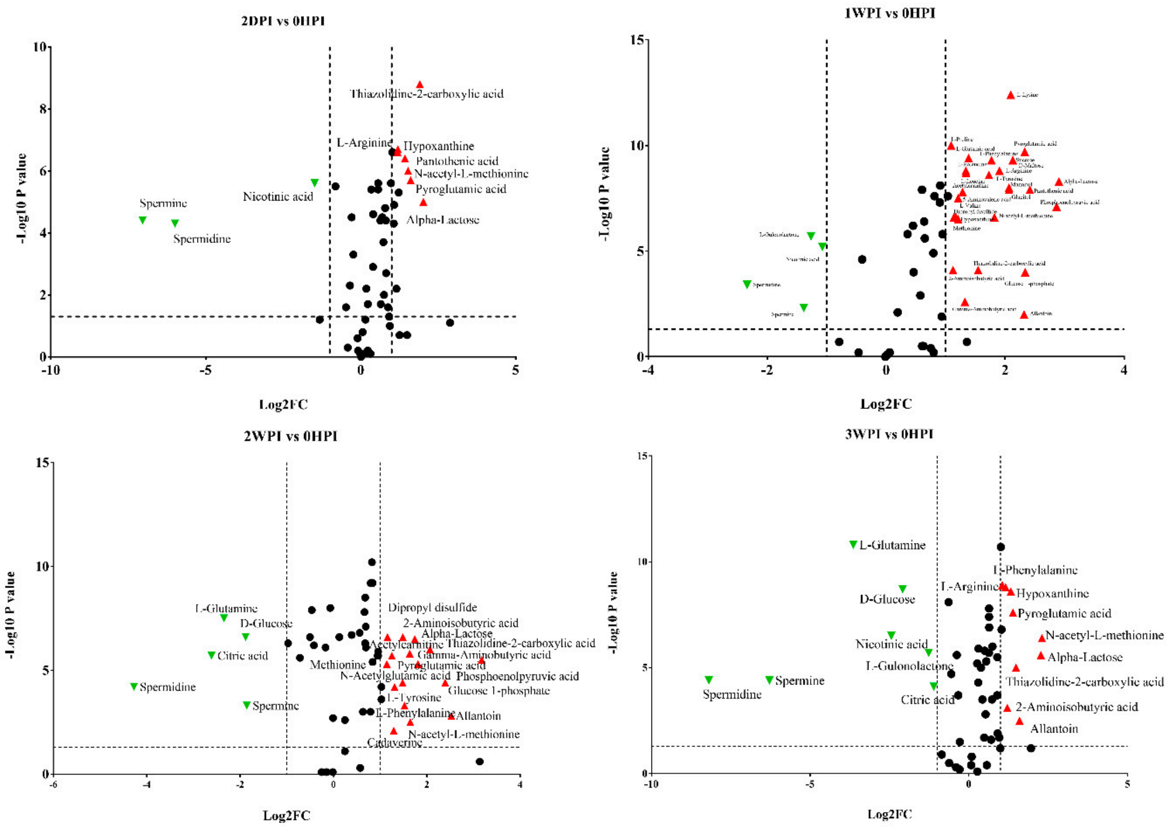

Figure S2. Volcano plots of all identified metabolites in Bm5 cells at different time points of CrPV infection: Comparison with uninfected cells. In the volcano plot, the log scaled value of fold change (*x*-axis) is plotted against the –log 10 *p*-value from multiple *t*-test analysis (*y*-axis). The dashed lines delineate metabolites with fold change > 2 and *p*-value < 0.05. Compound identities are displayed for both up regulated (right side; red upward triangle) or down regulated (left side; green downward triangle) metabolites. Different volcano plots compare different time points after CrPV infection (5 repeats of each).

Table S1. List of identified metabolites in silkworm-derived Bm5 cells upon CrPV infection.

Compound name	Formula	Adducts	Monoisotopic mass	Theoretical *m*/*z*-value	Average measured *m*/*z*-value	RT (min)	Class	HMDB	PubChem	KEGG	MSI level
L-Lysine	$C_6H_{14}N_2O_2$	$[M+H]^+$	146.1055	147.1128	147.1129471	0.90	Amino acids	HMDB0000182	5962	C00047	Tier1
L-Arginine	$C_6H_{14}N_4O_2$	$[M+H]^+$	174.1117	175.11895	175.1188507	0.81	Amino acids	HMDB0000517	6322	C00062	Tier1
L-Histidine	$C_6H_9N_3O_2$	$[M+H]^+$	155.0695	156.07675	156.0768433	0.78	Amino acids	HMDB0000177	6274	C00135	Tier1
Sucrose	$C_{12}H_{22}O_{11}$	$[M-H]^-$	342.1162	341.10893	341.1102944	1.02	Carbohydrates	HMDB0000258	5988	C00089	Tier1
L-Asparagine	$C_4H_7NO_4$	$[M+H]^+$	133.0375	134.04478	134.0447388	0.90	Amino acids	HMDB0000168	6267	C00152	Tier1
L-Serine	$C_3H_7NO_3$	$[M+H]^+$	105.0426	106.04987	106.0500821	0.90	Amino acids	HMDB0000187	5951	C00065	Tier1
L-Aspartic acid	$C_4H_7NO_4$	$[M+H]^+$	133.0375	134.04478	134.0447388	0.90	Amino acids	HMDB0000191	5960	C00049	Tier1
L-Glutamine	$C_5H_{10}N_2O_3$	$[M+H]^+$	146.0691	147.07642	147.0762787	0.89	Amino acids	HMDB0000641	5961	C00064	Tier1
L-Glutamic acid	$C_5H_9NO_4$	$[M+H]^+$	147.0532	148.06043	148.0603027	0.90	Amino acids	HMDB0000148	33032	C00025	Tier1
4-Hydroxyproline	$C_5H_9NO_3$	$[M+H]^+$	131.0582	132.06552	132.0654907	0.89	Amino acids	HMDB0000725	5810	C01157	Tier1
L-Cysteine	$C_3H_7NO_2S$	$[M+H]^+$	121.0198	122.02703	122.0275181	1.00	Amino acids	HMDB0000574	5862	C00097	Tier1
D-Fructose	$C_6H_{12}O_6$	$[M-H]^-$	180.0634	179.05611	179.0566293	1.00	Carbohydrates	HMDB0000660	439709	C02336	Tier1
D-Glucose	$C_6H_{12}O_6$	$[M+Na]^+$	202.5492	203.05261	203.0523071	0.90	Carbohydrates	HMDB0000122	5793	C00031	Tier1
Dipropyl disulfide	$C_6H_{14}S_2$	$[M+H]^+$	150.0537	151.06097	151.0615484	16.80	Organic disulfides	HMDB0031472	12377	C08373	Tier1
Gluconic acid	$C_6H_{11}NaO_7$	$[M+H]^+$	218.0402	219.04752	219.0471497	1.10	Carbohydrates	HMDB0000625	10690	C00257	Tier1
2-amino-isobutyric acid	$C_4H_9NO_2$	$[M+H]^+$	103.0633	104.07061	104.0708849	0.95	Amino acids	HMDB0001906	6119	C03665	Tier1
Alpha-Lactose	$C_{12}H_{22}O_{11}$	$[M+H]^+$	342.1162	343.12349	343.124448	0.90	Carbohydrates	HMDB0000186	84571	C00243	Tier1
D-Maltose	$C_{12}H_{22}O_{11}$	$[M-H]^-$	342.1162	341.10893	341.1099533	1.00	Carbohydrates	HMDB0000163	10991489	C00208	Tier1
L-Gulonolactone	$C_6H_{10}O_6$	$[M+H]^+$	178.0477	179.05501	179.0547791	0.92	Lactones	HMDB0003466	439373	C01040	Tier1
D-Galactose	$C_6H_{12}O_6$	$[M-H]^-$	180.0634	179.05611	179.0566293	1.00	Carbohydrates	HMDB0000143	439357	C00984	Tier1
L-Proline	$C_5H_9NO_2$	$[M+H]^+$	115.0633	116.07061	116.0707474	1.00	Amino acids	HMDB0000162	145742	C00148	Tier1
L-Threonine	$C_4H_9NO_3$	$[M+H]^+$	119.0582	120.06552	120.0657196	0.90	Amino acids	HMDB0000167	6288	C00188	Tier1
Thiazolidine-2-carboxylic acid	$C_4H_7NO_2S$	$[M+H]^+$	133.0198	134.02703	134.0271606	1.20	Carboxylic acids & derivatives	-	42486	-	Tier1

L-Valine	$C_5H_{11}NO_2$	$[M+H]^+$	117.0790	118.08626	118.086441	1.00	Amino acid	HMDB0000883	6287	C00183	Tier1
Gamma-Aminobutyric acid	$C_4H_9NO_2$	$[M+H]^+$	103.0633	104.07061	104.0707661	0.90	Carboxylic acids & derivatives	HMDB0000112	119	C00334	Tier1
5-Aminovaleric acid	$C_5H_{11}NO_2$	$[M+H]^+$	117.0790	118.08626	118.0865173	1.00	Amino acids	HMDB0003355	138	C00431	Tier1
Imidazolepropionic acid	$C_6H_8N_2O_2$	$[M+H]^+$	140.0586	141.06585	141.0659911	0.98	Imidazoles	HMDB0002271	70630	-	Tier1
Acetylcarnitine	$C_9H_{17}NO_4$	$[M+H]^+$	203.1158	204.12303	204.1230316	1.50	Fatty acid esters	HMDB0000201	1	C02571	Tier1
Urocanic acid	$C_6H_6N_2O_2$	$[M+H]^+$	138.0429	139.0502	139.0501404	1.43	Imidazoles	HMDB0000301	736715	C00785	Tier1
D-Ribose	$C_5H_{10}O_5$	$[M-H]^-$	150.0528	149.04555	149.0462505	1.50	Carbohydrates	HMDB0000283	5779	C00121	Tier1
D-Arabinose	$C_5H_{10}O_5$	$[M-H]^-$	150.0528	149.04555	149.0462505	1.00	Carbohydrates	HMDB0029942	66308	-	Tier1
Methionine	$C_5H_{11}NO_2S$	$[M+H]^+$	149.0511	150.05833	150.0582733	1.50	Amino acids	HMDB0000696	6137	C00073	Tier1
Pyro-Glutamic acid	$C_5H_7NO_3$	$[M+H]^+$	129.0426	130.04987	130.0498657	1.66	Amino acids	HMDB0000267	7405	C01879	Tier1
N-acetyl-Glutamic acid	$C_7H_{11}NO_5$	$[M-H]^-$	189.0637	188.05645	188.0556335	1.81	Amino acids	HMDB0001138	185	C00624	Tier1
L-Tyrosine	$C_9H_{11}NO_3$	$[M+H]^+$	181.0739	182.08117	182.0810547	2.00	Amino acids	HMDB0000158	6057	C00082	Tier1
L-Isoleucine	$C_6H_{13}NO_2$	$[M+H]^+$	131.0946	132.10191	132.1018524	2.10	Amino acids	HMDB0000172	6306	C00407	Tier1
L-Leucine	$C_6H_{13}NO_2$	$[M+H]^+$	131.0946	132.10191	132.1019592	2.30	Amino acids	HMDB0000687	6106	C00123	Tier1
L-Phenylalanine	$C_9H_{11}NO_2$	$[M+H]^+$	165.0790	166.08626	166.0860291	4.20	Amino acids	HMDB0000159	6140	C00079	Tier1
Pantothenic acid	$C_9H_{17}O_5N$	$[M+H]^+$	218.6068	219.11012	219.1107773	7.36	Carboxylic acids & derivatives	HMDB0000210	988	C00864	Tier1
N-acetyl-L-methionine	$C_7H_{13}NO_3S$	$[M+H]^+$	191.0616	192.06889	192.0688324	6.09	Amino acids	HMDB0011745	6180	C02712	Tier1
3-Methyl-2-cyclohexen-1-one	$C_7H_{10}O$	$[M+H]^+$	110.0732	111.08044	111.0807685	8.43	Ketones	HMDB0031541	14511	-	Tier1
Glucitol	$C_6H_{14}O_6$	$[M+H]^+$	182.0790	183.08631	183.0868593	1.00	Carbohydrates	HMDB0000247	5780	C00794	Tier1
Glycerol	$C_3H_8O_3$	$[M+H]^+$	92.0473	93.05462	93.05489916	1.00	Carbohydrates	HMDB0000131	753	C00116	Tier1
Mannitol	$C_6H_{14}O_6$	$[M+H]^+$	182.0790	183.08631	183.0865546	0.92	Carbohydrates	HMDB0000765	6251	C00392	Tier1
Pipecolic acid	$C_6H_{11}NO_2$	$[M+H]^+$	129.0790	130.08626	130.0863381	1.50	Carboxylic acids & derivatives	HMDB0000070	849	C00408	Tier1
Nicotinic acid	$C_6H_5NO_2$	$[M+H]^+$	123.0320	124.0393	124.0394287	1.45	Pyridines and derivatives	HMDB0001488	938	C00253	Tier1
Citric acid	$C_6H_8O_7$	$[M+H]^+$	192.0270	193.03428	193.0346661	1.38	Carboxylic acids & derivatives	HMDB0000094	311	C00158	Tier1

Phenylpropionic acid	$C_9H_{10}O_2$	[M-H]⁻	150.0681	149.0608	149.0613813	9.50	Phenylpropanoic acids	-	107	-	Tier1
Spermidine	$C_7H_{19}N_3$	[M+H]⁺	145.1579	146.16517	146.1651764	0.70	Amines	HMDB0001257	1102	C00315	Tier1
Spermine	$C_{10}H_{26}N_4$	[M+H]⁺	202.2157	203.22302	203.2227631	0.70	Amines	HMDB0001256	1103	C00750	Tier1
Cadaverine	$C_5H_{14}N_2$	[M+H]⁺	102.1157	103.12298	103.1231862	0.70	Amines	HMDB0002322	273	C01672	Tier1
Glucose 1-phosphate	$C_6H_{11}O_9PNa_2$	[M+H]⁺	303.9936	305.00088	305.0003052	0.80	Carbohydrates	HMDB0001586	439165	C00103	Tier1
Glycerol 1-phosphate	$C_3H_9O_6P$	[M+H]⁺	172.0137	173.02095	173.0209045	1.02	Glycerophospholipids	HMDB0000126	439162	C00093	Tier1
Allantoin	$C_4H_6N_4O_3$	[M+H]⁺	158.0440	159.05127	159.0517472	0.98	Azolidines	HMDB0000462	204	C01551	Tier1
Glyceraldehyde	$C_3H_6O_3$	[M+H]⁺	90.0317	91.03897	91.03953936	0.95	Carbohydrates	HMDB0001051	751	C02154	Tier1
Phosphoenolpyruvic acid	$C_3H_4NaO_6P$	[M+H]⁺	189.9643	190.9716	190.9719819	0.95	Phosphate esters	HMDB0000263	1005	C00074	Tier1
Hypoxanthine	$C_5H_4N_4O$	[M+H]⁺	136.0385	137.04579	137.0458069	1.57	Purines and purine derivatives	HMDB0000157	790	C00262	Tier1
Tyramine	$C_8H_{11}NO$	[M+H]⁺	137.0835	138.09013	138.0904205	1.09	Phenethylamines	HMDB0000306	5610	C00483	Tier1
Cyclohexylamine	$C_6H_{13}N$	[M+H]⁺	99.1048	100.11208	100.112394	3.16	Cyclohexylamines	HMDB0031404	7965	C00571	Tier1

Metabolites identified in Bm5 cells upon CrPV infection were listed. Adducts: protonated or deprotonated molecular ions, $[M+H]^+$, $[M-H]^-$, and $[M+Na]^+$. RT min: Retention time (min); Matched ID for each database (HMDB: Human Metabolome Database; Pubchem: https: // pubchem .ncbi.nlm.nih.gov; KEGG: Kyoto Encyclopedia of Genes and Genomes) were provided; MSI level, metabolomics standard initiative level of identification.
